# Monitoring of Bactericidal Effects of Silver Nanoparticles Based on Protein Signatures and VOC Emissions from *Escherichia coli* and Selected Salivary Bacteria

**DOI:** 10.3390/jcm8112024

**Published:** 2019-11-19

**Authors:** Fernanda Monedeiro, Paweł Pomastowski, Maciej Milanowski, Tomasz Ligor, Bogusław Buszewski

**Affiliations:** 1 Department of Environmental Chemistry and Bioanalytics, Faculty of Chemistry, Nicolaus Copernicus University, 87-100 Toruń, Poland; fernandamonedeiro@usp.br (F.M.); pawel_pomastowski@wp.pl (P.P.); milanowski.maciej@gmail.com (M.M.); msd2501@chem.uni.torun.pl (T.L.); 2 Interdisciplinary Centre of Modern Technologies, Nicolaus Copernicus University, 87-100 Toruń, Poland; 3 Department of Chemistry, Faculty of Philosophy, Science and Letters of Ribeirão Preto, University of São Paulo, Ribeirão Preto CEP 14040-901, Brazil

**Keywords:** bacteria, HS-SPME-GC-MS, MALDI-TOF MS, silver nanoparticles, VOCs

## Abstract

*Escherichia coli* and salivary *Klebsiella oxytoca* and *Staphylococcus saccharolyticus* were subjected to different concentrations of silver nanoparticles (AgNPs), namely: 12.5, 50, and 100 µg mL^−1^. Matrix-assisted laser desorption/ionization–time-of-flight mass spectrometry (MALDI-TOF MS) spectra were acquired after specified periods: 0, 1, 4, and 12 h. For study of volatile metabolites, headspace solid-phase microextraction coupled to gas chromatography/mass spectrometry (HS-SPME-GC-MS) was employed—AgNPs were added to bacteria cultures and the headspace was analyzed immediately and after 12 h of incubation. Principal components analysis provided discrimination between clusters of protein profiles belonging to different strains. Canonical correlation, network analysis, and multiple linear regression approach revealed that dimethyl disulfide, dimethyl trisulfide, 2-heptanone, and dodecanal (related to the metabolism of sulfur-containing amino acids and fatty acids synthesis) are exemplary molecular indicators, whose response variation deeply correlated to the interaction with bacteria. Therefore, such species can serve as biomarkers of the agent’s effectiveness. The present investigation pointed out that the used approaches can be useful in the monitoring of response to therapeutic treatment based on AgNPs. Furthermore, biochemical mechanisms enrolled in the bactericidal action of nanoparticles can be applied in the development of new agents with enhanced properties.

## 1. Introduction

Silver nanoparticles (AgNPs) are widely used in a growing number of industrial and medical applications, such as electronics, food industry, paints, clothing, cosmetics, and medical devices [1,2]. They are well-known antimicrobial agents and their antimicrobial activity against bacteria is attributed to their high reactivity with proteins and initiation of structural changes in the cell wall and membrane. Silver nanoparticles can interact with SH-groups of amino acids and hence inhibit protein synthesis and function. Also, uptake of free silver ions followed by disruption of ATP production and DNA replication is one possible mechanism of toxicity. As a consequence, it leads to inhibition of vital functions and cell death [3,4]. Possible mechanisms of function of silver nanoparticles are described in many publications, however, there are still gaps in the understanding of this phenomenon [4]. Effectiveness of function of AgNPs was investigated by means of various techniques including well and disc diffusion methods and flow cytometry [5,6,7,8,9]. In the current study, we employed matrix-assisted laser desorption/ionization–time-of-flight mass spectrometry (MALDI-TOF MS), which has emerged as a powerful technique for identification of microorganisms [10,11] and for investigation of, for instance, bacterial drug resistance [12]. Protein profiles obtained within the mass range of 2000 to 20,000 Da using MALDI-TOF MS can reflect many physiological states of bacteria [10]. Ag nanoparticles have a dual function in MALDI-TOF MS analyzes, they can serve as a MALDI matrix for enhanced detection of bacteria or as a bactericidal agent. A critical limiting threshold concentration, which governs whether AgNPs would function as an affinity probe or would express bactericidal property, was investigated in the study of Gopal et al. (2011) [13]. Authors conducted their studies using two model bacterial strains: *Escherichia coli* and *Serratia marcescens*. They stated that critical concentration of affinity probes (CCAP) for silver nanoparticles was 1 mL L^−1^ in the case of *E. coli* and 0.5 mL L^−1^ for *S. marcescens*. Above these concentrations, AgNPs became bactericidal for tested bacteria.

Saliva is a biological matrix with promising applications in clinical settings. Its rapid, non-invasive, and cost-effective collection, easy storage and transportation are advantages over other specimens [14,15]. Like serum, saliva contains hormones, antibodies, growth factors, enzymes, microbes, and their products. Consequently, saliva can be considered as a “mirror” of the body for diagnostic purposes of (inter alia) oral diseases [16]. Volatile organic compounds (VOCs) are one of salivary constituents that can indicate various oral conditions, such as halitosis, periodontal disease, lung cancer, and celiac disease [17]. Gas chromatography–mass spectrometry (GC-MS) is a technique which allows detection of VOCs from different niches, including breath, tissues, saliva, or bacteria [18,19,20,21,22,23]. Solid phase microextraction (SPME) in headspace (HS) variant is a commonly used method for extraction and enrichment of VOCs from many biological matrices [24,25,26]. Also, VOCs from bacterial strains were analyzed using gas chromatography–mass spectrometry technique. For example, the effect of applied growth medium on emission of volatiles from *E. coli* was investigated in the work of Ratiu et al. (2017) [27]. *Streptococcus pneumoniae* and *Haemophilus influenzae* cultures were identified to find characteristic volatile biomarkers of these bacteria associated with community-acquired pneumonia (CAP) [28]. *Staphylococcus aureus* and *Pseudomonas aeruginosa* were investigated by Filipiak et al. (2012) [29] to find volatile organic compounds from bacteria most frequently found in ventilator-associated pneumonia (VAP) patients. Headspace samples from bacteria were collected and preconcentrated on multibed sorption tubes and analyzed by GC-MS. Once the investigation of bacterial profiles of molecular species can be related with the presence of a specific strain and its metabolic behavior, the features regarding the activity of bactericidal agents can be measured and thoroughly investigated employing the comprehensive evaluation of these profiles.

The aim of the present work is to provide correlated relationships between expressed proteins and volatile metabolites, as a form to assess bactericidal agent performance. Moreover, such knowledge can contribute for elucidation of action mechanism of silver nanoparticles and how they can influence the physiological state of selected bacteria. Growth curves of *E. coli*, *Klebsiella oxytoca*, and *Staphylococcus saccharolyticus* were prepared, then, MALDI-TOF MS technique was employed to obtain proteins profiles of these bacteria and to investigate the addition of selected levels of AgNPs on them. Furthermore, in order to assess alterations in the produced bacterial volatile metabolites, VOCs were investigated by means of HS-SPME-GC-MS, for strains in the unstressed form and after their treatment with silver nanoparticles.

## 2. Experimental Section

### 2.1. Instruments

The ultrafleXtreme MALDI-TOF/TOF mass spectrometer (Bruker Daltonik, Bremen, Germany) equipped with a modified Nd:YAG laser (Smartbeam IITM) operating at the wavelength of 355 nm and the frequency of 2 kHz was used to acquire spectra from strains of bacteria by means of two methods and to investigate the interactions between silver nanoparticles and bacterial cells. Optical density (OD) measurements were performed with a DEN-1B Densitometer (Biosan, Riga, Latvia). VITEK^®^ 2 Compact system (bioMérieux, Marcy l’Etoile, France) was employed for identification of salivary bacteria. The GC-MS analyses were carried out using an Agilent 6890A gas chromatograph coupled to an Agilent 5975 Inert XL MSD mass spectrometer (both from Agilent Technologies, Santa Clara, CA, USA). The system was equipped with a Rtx^®^-5MS w/Integra Guard 30 m × 0.25 mm × 0.25 µm column (Restek Corporation, Bellefonte, PA, USA). Extractions of volatile organic compounds were performed using 65 µm polydimethylsiloxane (PDMS)/divinylbenzene (DVB) fiber (Supelco, Bellefonte, PA, USA). Incubating Microplate Shaker (VWR International, Radnor, PA, USA) was used for incubation of headspace vials with bacterial content.

### 2.2. Materials

Water LC-MS Chromasolv, ethanol, acetonitrile (ACN), trifluoroacetic acid (TFA), formic acid, and isopropanol were purchased from Sigma Aldrich (Steinheim, Germany). Ultra-pure water from a Milli-Q water system (Millipore, Bedford, MS, USA) was used throughout the work.

All chemicals for MALDI-MS analyses were supplied at the highest commercially available purity from Fluka Feinchemikalien GmbH (part of Sigma Aldrich). Polished steel targets (Bruker Daltonik) were used for sample deposition. α-cyano-4-hydroxycinnamic acid (HCCA; Sigma Aldrich) was employed as a matrix for MALDI analyses (dried droplet method). Bruker bacterial test standard (BTS) was used for external calibration (Bruker Daltonik).

Then, 15 mL sterile polypropylene tubes (ISOLAB, Wertheim, Germany) were used for collection of oral fluid. For identification of salivary bacteria strains, we used 0.45% saline and VITEK^®^ 2 Compact ID Cards for Gram-negative (GN), Gram-positive (GP), and anaerobe corynbacteria (ANC) (bioMérieux). Headspace screw top 20 mL clear vials and magnetic polytetrafluoroethylene (PTFE)/Sil screw caps for headspace vials, 18 mm thread, were purchased from Agilent Technologies.

Three media were used for bacteria cultivation: tryptic soy broth (TSB; Soybean-Casein Digest Medium; Bacto, Sydney, Australia), Mueller Hinton (MH) broth, and M9 (both from Sigma Aldrich). Glucose used as an additive to the minimal medium M9 was purchased from Avantor Performance Materials (Gliwice, Poland). Detailed information regarding media technical aspects can be found elsewhere [30].

The silver nanoparticles used in this study were synthesized in our laboratory and physicochemical characterized previously by Railean-Plugaru et al. (2016) [31]. They were synthesized by the biological method. In comparison with the chemically synthesized one, it was found to be naturally coated with organic deposit, therefore, considered as a biocolloid. The biologically synthesized nanocomposites were tested against 7 different bacterial strains and the antimicrobial activity was found to be dependent on the silver nanocomposite concentrations [9,31].

Two bacterial strains used during investigations, namely: *Escherichia coli* ATCC 25922 and salivary *Klebsiella oxytoca* ATCC 13182 were obtained from POL-AURA (Dywity, Poland). The strain of *Staphylococcus saccharolyticus* was isolated from the saliva of a healthy human oral cavity and identified using VITEK^®^ 2 Compact system, as described by Buszewski et al. (2017) [32].

### 2.3. Growth Curves of Bacteria

In order to draw the growth curves of the three bacteria, the OD of samples was measured using DEN-1B Densitometer, which provided results in the unit of McFarland (McF). First, three test tubes were filled with 4.8 mL of M9 medium and sterilized by autoclaving. Once the test tubes cooled down, 0.2 mL of prefiltered 10% (m/v) solution of glucose, which served as a source of carbon for bacteria, was added to each tube—the concentration of glucose in the obtained 5 mL of solution was 0.4% (m/v). The content of all test tubes was vortexed for 30 s and the OD of obtained blanks was measured using DEN-1B Densitometer. Then, three loopfuls of bacterial cells were suspended in 1 mL of saline solution to prepare inoculum. Bacterial suspension was thoroughly vortexed for 30 s and the test tubes were inoculated under sterile conditions using 100 µL of the obtained inoculum. Immediately after inoculation, OD at t_0_ was determined. Subsequent measurements were performed at t_2_, t_4_, t_6_, t_8_, t_23_, t_25_, t_27_, t_29_, and t_31_, corresponding to 2, 4, 6, 8, 23, 25, 27, 29, and 31 h of incubation at 37 °C. Growth profiles were assessed for selection of cultivation times to be used in further assays. Such cultivation periods were aimed to refer to the stationary phase of these bacteria, because in this stage the ratio alive/dead is rather constant, thus, the changes observed in the molecular profiles could be ascribed to a metabolic response to the added stressing agent, minimizing the contribution of metabolic alterations due to the growth process.

### 2.4. MALDI-TOF MS Analysis

MALDI-TOF MS spectra were recorded manually in linear positive mode within *m*/*z* range of 3000–30000 and applying the acceleration voltage of 25 kV. All mass spectra were acquired and processed with the dedicated software: flexControl and flexAnalysis, respectively (both from Bruker). Two following experiments with MALDI MS technique were conducted in triplicate during the study.

#### 2.4.1. Comparison of Sample Preparation Methods

The first experiment concerned a comparison between two sample preparation protocols for microorganism profiling, according to the instructions of mass spectrometer manufacturer. The “DIRECT” procedure consists in direct smearing of a sample onto a MALDI target, whereas “EXTRACTION” involves extraction of proteins from a sample with ethanol and formic acid. The HCCA matrix (10 mg mL^−1^) was prepared in a standard solvent (50% acetonitrile, 47.5% water, 2.5% trifluoroacetic acid). The “DIRECT” procedure was as follows: (i) direct smearing of a small amount of biological material (barely visible) using pipette tips onto a sample spot of a polished steel target, (ii) overlaying the biological material with 1 µL of HCCA matrix solution, (iii) allowing the sample spot to air dry before analysis. The above-mentioned biological material was a precentrifuged (13,000 rpm/RCF = 15,871× *g* for 2 min) bacterial pellet obtained from 1.5 mL of a liquid culture. For our purpose, we applied three different media (5 mL of TSB, MH, or M9) which were inoculated using a loopful with three bacterial strains and incubated at 37 °C for 24 h. Moreover, we performed OD measurements of a medium alone, a sample after inoculation, and a sample after incubation (next day in the morning). In addition, three types of media alone (TSB, MH, and M9) served as blanks. The “EXTRACTION” protocol was used in the following way: (i) 300 µL of water was transferred into an eppendorf tube containing the biological material and mixed; (ii) then 900 µL of 100% ethanol was added to the tube and mixed thoroughly; (iii) this was followed with centrifugation at 13,000 rpm/RCF = 15,871× *g* for 2 min and decanting the supernatant; (iv) centrifugation was continued for further 2 min and residual ethanol was removed from the pellet using a pipet; (v) subsequently, 5 µL of 70% formic acid was added to the pellet and mixed thoroughly by pipetting and by vortexing; (vi) 5 µL of acetonitrile was added to the tube and mixed carefully; (vii) the whole was centrifuged at 13,000 rpm/RCF = 15,871× *g* for 2 min and 1 µL of the supernatant was spotted onto a polished steel target; (viii) the sample was covered with 1 µL of HCCA matrix solution as soon as the sample spot had dried out; (ix) finally, the sample spot was allowed to air dry before analysis. The biological material for “EXTRACTION” method was a precentrifuged (13,000 rpm/RCF = 15,871× *g* for 2 min) bacterial pellet obtained from 1.5 mL of a liquid culture with MH medium. In this part of the experiment we used only MH medium (5 mL) inoculated using a loopful with three bacteria and incubated at 37 °C for 24 h. The samples were smeared onto a MALDI target in triplicate. Extract from pure MH was used as a blank in this step.

#### 2.4.2. Influence of AgNPs on MALDI-TOF MS Profiles of the Selected Bacteria

A total of 150 mL of MH medium was prepared and shared between three Erlenmeyer flasks (50 mL in each). After sterilization, each portion of medium was inoculated with two loopfuls of bacterial cells (approximately 1 × 10^6^ of cells) of *E. coli*, *K. oxytoca*, and *S. saccharolyticus* individual strains and placed into a shaker at 37 °C for 8, 15, or 18 h, depending on strain type (based on growth curves experiments). Then, under sterile conditions, nine sterilized test tubes received 10 mL of liquid culture each. Immediately, silver nanoparticles were added and vortexed to obtain the final silver concentration of 12.5, 50, and 100 µg mL^−1^. After that, OD measurements were performed. Next, all test tubes with their content were placed into an incubator at 37 °C for a specified period of time, namely: 0, 1, 4, and 12 h, then 1.5 mL of each solution was transferred to an Eppendorf tube and centrifuged at 13,000 rpm/RCF = 15,871× *g* for 2 min. The supernatant was removed and the bacterial pellet was subjected to the “EXTRACTION” technique (treatment of a sample using ethanol and formic acid).

### 2.5. Gas Chromatography–Mass Spectrometry (GC-MS)

A total of 4 mL of sterilized MH broth was inoculated with 100 µL of a bacterial suspension (already cultured bacteria, under 37 °C for 8, 15, or 18 h, depending on strain type) of *E. coli, K. oxytoca*, and *S. saccharolyticus* in the medium (100 µL of pure medium was used as a blank). Before application of AgNPs, OD of the inoculum was always measured and was approximately 1.5 McF. Immediately, silver nanoparticles were added to obtain the final concentration of 12.5 and 50 µg mL^−1^. All four headspace vials (three with bacteria and one with a blank) were placed into an incubator at 37 °C with continuous shaking. Then, the vials were taken out immediately (t = 0) and after 12 h (t = 12). VOCs were extracted at 37 °C for 45 min, using 65 µm PDMS/DVB fiber. All GC-MS experiments were done in triplicate. Helium with a flow rate of 1.1 mL min^−1^ was the carrier gas and temperature of the split–splitless injector was set at 240 °C. The oven temperature program was as follows: The initial temperature of 40 °C was kept for 3 min, then ramped at 10 °C min^−1^ to 300 °C and kept at this last temperature for 5 min. Spectra acquisition was performed within *m*/*z* range of 30–300 with electron ionization (EI) at 70 eV; both the ion source and the transfer line temperature was set at 250 °C.

### 2.6. Data Processing and Statistical Analysis

Part of the data processing and statistical analysis was performed in R environment, using RStudio v.1.1.463 console. GC-MS raw data (CDF format) was processed using the “xcms” package, peaks were detected in the shift region of 10 ppm, applying the “centWave” method. As result, a database displaying retention time and corresponding area for extracted ion chromatogram (EIC) was obtained. Peaks arising from medium blanks in different incubation times were subtracted from the related bacterial samples. Peak identification was performed using NIST11, for each peak detected belonging to the same retention time—a match factor of at least 750/1000 was considered. From MALDI-TOF MS data, the 100 most intense and corresponding signals were annotated and ion database was created manually.

A comparison between MALDI-TOF MS raw spectra (mzXML format) obtained from bacterial extract before and after AgNPs addition was performed by calculation of the spectrum similarity score (SSS), with usage of “OrgMassSpecR” package in default mode. Heatmaps based on the area of ions, associated with hierarchical cluster analysis using the Spearman method, were built employing “gplots” package.

Canonical correlation analysis (CCA) is a multivariate ordination analysis used to provide sample correlations between two sets of variables. This method was employed to relate the variation in the fold-change of VOCs and proteins ions after the treatment with nanoparticles. The analysis was conducted using representative features (*p* < 0.05, indicated by Mann–Whitney test), “vegan” package was applied to run CCA function.

Network analysis was performed using “igraph” package, in order to provide visualization of the connections between both sets of data. Spearman’s coefficients were used to produce the edges between nodes representing protein ions and VOCs, which presented statistically relevant (*p* < 0.05) change in their response after the addition of nanoparticles.

With the usage of IBM SPSS Statistics v.24, Spearman correlation coefficient was calculated between triplicates to verify the reproducibility of the obtained profiles. Principal components analysis (PCA) was carried out to assess the distribution of MALDI-TOF MS profiles according to strain. Mann–Whitney U test was performed to indicate discriminating features. Linear regression was used to create a model able to predict the extension of AgNPs interaction with bacteria. Detailed information regarding used R packages and input formats, as well as employed databases are available in Supplementary Materials.

## 3. Results

### 3.1. Determination of Growth Curves

Figure S1 depicts growth curves of three selected bacteria grown in minimal medium M9 with glucose as a source of carbon. Based on the assessed curves, further experiments were conducted using 8, 15, or 18 h of cultivation of E. coli, K. oxytoca, and S. saccharolyticus, respectively. Bacteria at the given times were at the beginning of the stationary phase. Previous experiments reported growth profiles of the selected bacteria using MH medium [33,34,35].

### 3.2. Comparison of Procedures: Direct Smearing Versus Extraction and Medium Selection

**Figure 1:**
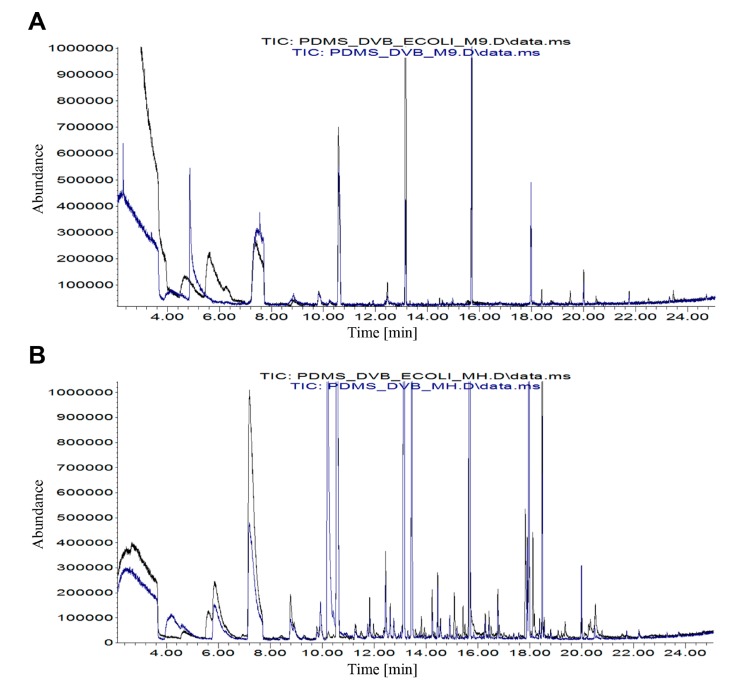
Comparison of overlaid gas chromatography–mass spectrometry (GC-MS) chromatograms obtained for (**A**) *E.coli* bacterium + M9 medium (black) versus pure M9 medium (blue) and (**B**) *E.coli* bacterium + Mueller Hinton (MH) medium (black) versus pure MH medium (blue).

Straight and clear baseline with negligible signals recorded in the acquired spectra were seen for all tested growth media (M9, MH, and TSB) after using “DIRECT” and “EXTRACTION” (MH only) protocols (Figure S2). Figure S3 demonstrates an exemplary comparison of MALDI-TOF MS spectra of *E. coli* cultured in M9, MH, and TSB media, obtained using “DIRECT” method. The presented observations for *E. coli* are typical for our selected bacterial strains. M9 was found to be the most effective medium among the used growth media. The number of peaks reached almost 100. Moreover, the number of signals with signal-to-noise ratio (S/N) greater than or equal to 10 was 77. Only 33 and 34 signals with such defined S/N were recorded for MH and TSB medium, respectively. However, for further MALDI-TOF MS investigations concerning addition of silver nanoparticles, we selected MH medium, since it provided the best results in the simultaneously conducted GC-MS study (Figure 1).

**Figure 2:**
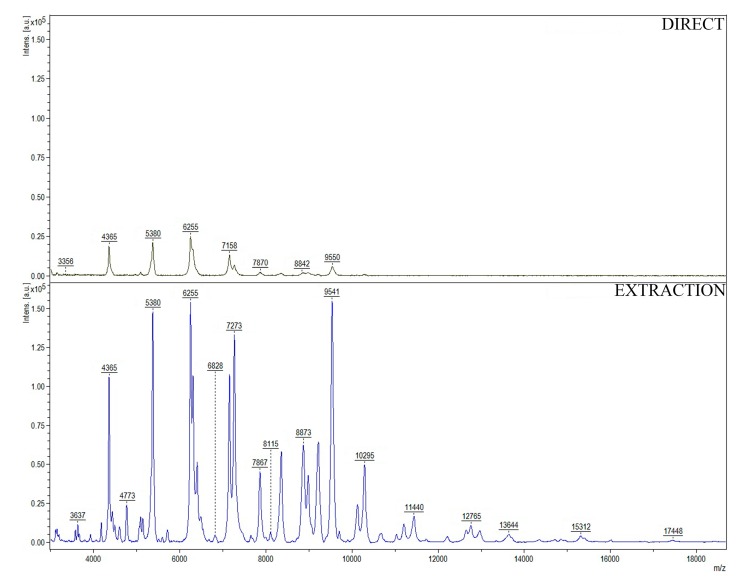
Representative (from triplicate experiment) matrix-assisted laser desorption/ionization–time-of-flight mass spectrometry (MALDI-TOF MS) spectra of *E. coli* in MH medium obtained using the “DIRECT” and “EXTRACTION” procedure.

Figure 2 shows a comparison between the two applied sample preparation techniques with the example of E. coli.

The differences between MALDI-TOF MS spectra include both the number of detected peaks and their intensity. For E. coli, in the “EXTRACTION” method, we recorded 54 signals with S/N greater than or equal 10. On contrary, for the “DIRECT” procedure, we found only 33 peaks fulfilling this requirement. Moreover, signals obtained for the “EXTRACTION” procedure were several times more intense than the values for the “DIRECT” technique. Similar observations were extended to the other studied bacteria. It indicates that sample treatment using ethanol and formic acid is far more efficient than the method consisting in direct smearing of a sample onto a MALDI target.

### 3.3. Influence of Silver Nanoparticles on MALDI-TOF MS Spectra and HS-SPME-GC-MS Chromatograms of the Selected Bacteria

To investigate the influence of AgNPs on MALDI-TOF MS spectra of the three model bacteria, the following tests were conducted. In this part of the study, the “EXTRACTION” technique was applied for sample preparation of each bacterial strain, as it was found to be superior to the “DIRECT” procedure in the previous experiment.

Regarding MALDI-TOF MS experiments, the prepared database displayed 1928 most relevant ions, ranging from 3013 to 17,449 *m*/*z*. In GC-MS data, ions addressed as belonging to medium, GC column material, or fiber coating were subtracted from the database, resulting in a total number of 281 computed ions, ranging from 32 to 298 *m*/*z*.

**Figure 3:**
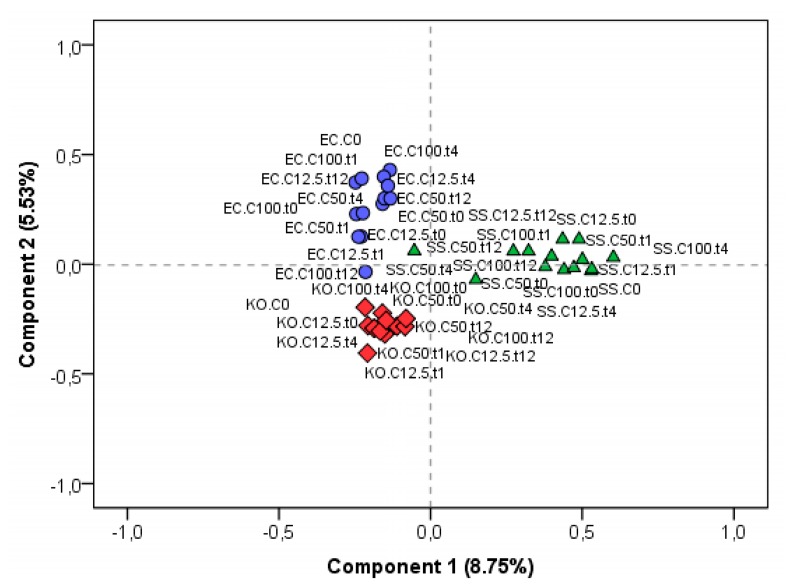
Principal component analysis (PCA) plot of protein ions profiles acquired by MALDI-TOF MS experiments, where diamonds, triangles, and circles represent *K. oxytoca* (KO), *S. saccharolyticus* (SS), and *E. coli* (EC), respectively.

Figure 3 presents the PCA score plot obtained for different protein ions profiles acquired under the mentioned experimental conditions, for each bacterial strain. Profiles belonging to an individual bacterial strain were clearly separated from others, highlighting the discriminative power of ribosomal profiles in bacteria identity. However, still being possible to observe that the performed assays were capable of significantly altering such profiles, in this case, the distinction between the 3 strains was explained by only 14% of the variance.

**Figure 4:**
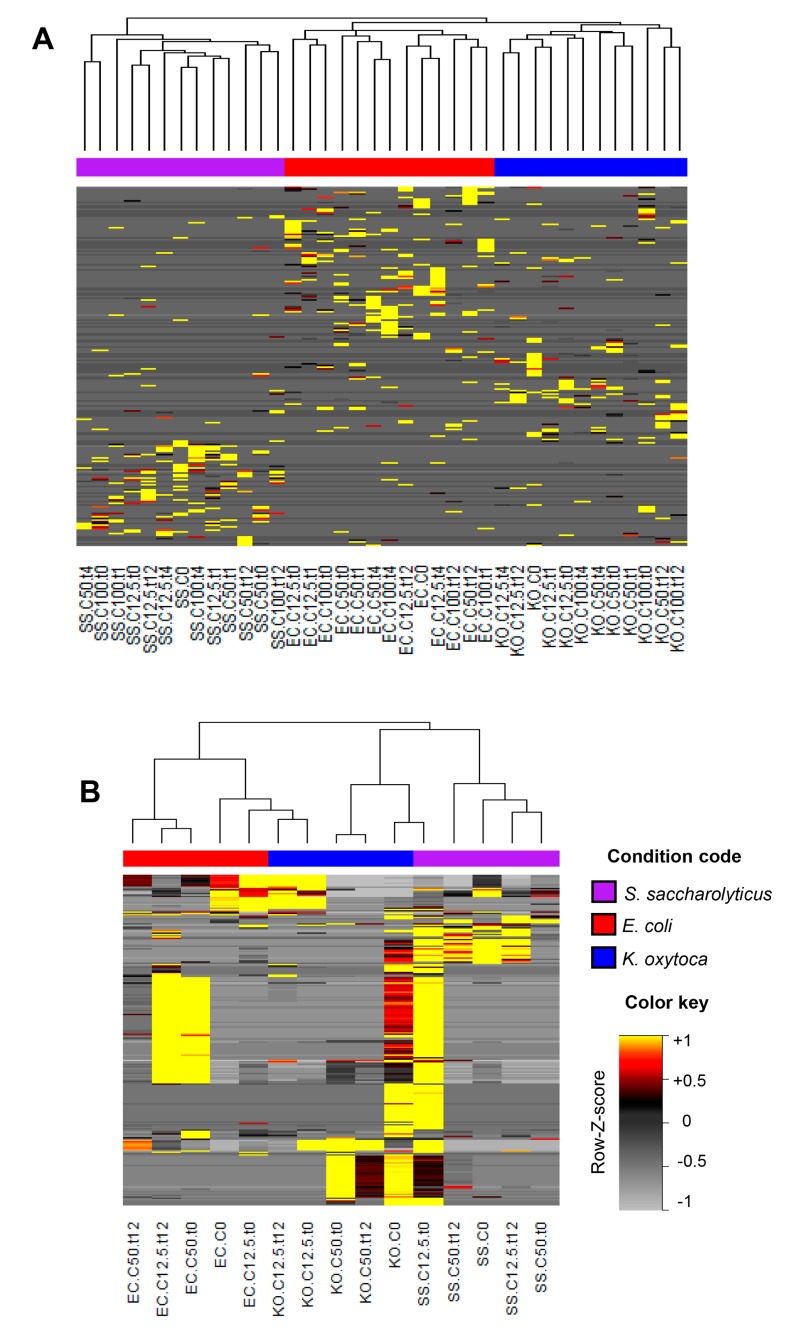
Heatmaps and hierarchical cluster analysis of protein ions (**A**) and volatile organic compound (VOC) profiles (**B**), based on ion areas extracted from MALDI-TOF MS spectra and GC-MS chromatograms, respectively; Horizontal axis = conducted assays, using different bacterial strains, vertical axis = ions organized in clusters.

Heatmaps referring to protein ions profiles (Figure 4A) and VOC profiles (Figure 4B) allow inspection of the distribution of ions detected in each experiment. Hierarchical cluster analysis evidencing the similarity between these individual profiles and segregation according to bacterial strain were observed in data extracted both by MALDI-TOF MS and GC-MS results. The ions constituting the profiles were grouped in clusters, which correspond to the main distribution behaviors identified along the samples.

**Figure 5:**
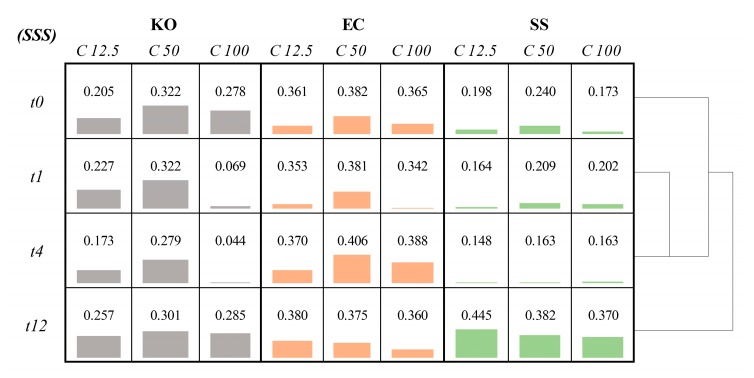
Matrix presenting average values of spectra similarity score (SSS), concerning comparison between unstressed spectrum and assays using silver nanoparticles (AgNPs) at 12.5, 50, and 100 µg mL^−1^, for periods of time 0, 1, 4, and 12 h. Gray, orange, and green bars refer to *K. oxytoca* (KO), *S. saccharolyticus* (SS), and *E. coli* (EC), respectively; C—concentration (µg mL^−1^); t—time (h).

In order to objectively quantify the overall changes in MALDI-TOF MS spectra after the addition of nanoparticles, the SSS parameter was assessed. The average SSSs calculated for each experiment are displayed in Figure 5. This parameter mathematically represents the average level of similarity between spectra acquired for unstressed strain and the same strain after supplementation with AgNPs. The clusters on the right side of the matrix are based on Euclidian distance and represent more similar behaviors during assay with nanoparticles.

For *K. oxytoca*, it can be seen that for concentration 100 µg mL^−1^, the biggest changes in profiles were obtained for 1 and 4 h after supplementation with AgNPs. After 12 h, an adaptation to the stressing agent is observable resulting in an increased spectrum similarity score to the original native strain. For lower concentrations (12.5 and 50 µg mL^−1^), *K. oxytoca* manifested stronger resistance to applied silver nanoparticles. *E. coli* was the most resistant for concentration 50 µg mL^−1^, bigger alternations were noticed for the remaining concentrations. On the other hand, *S. saccharolyticus* appeared to be the most susceptible to added AgNPs. This strain reacted the most in the first hours after supplementation of silver nanoparticles. The strain showed tolerance to stressing agent only after 12 h of influence, regardless of the used concentration. In a general view, the apex of interaction of AgNPs with intercellular portion can be addressed as the higher studied concentration (100 µg mL^−1^), associated to the incubation time of 4 h for *K. oxytoca* and *S. saccharolyticus* and 1 h for *E. coli*. Once 12 h of incubation was reached, an overall tendency to return to the untreated-like profile is observed for all strains.

**Figure 6:**
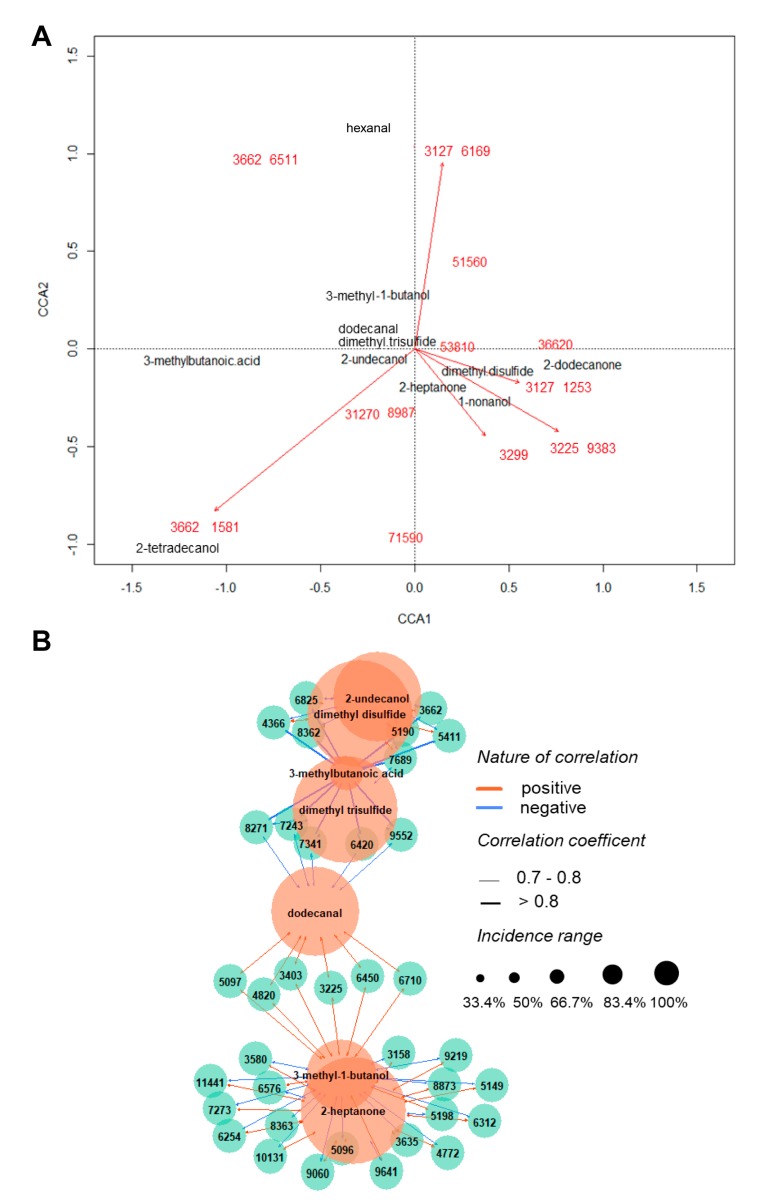
(**A**) Canonical correlation analysis (CCA) biplot showing the correspondence of the main protein ions (red) with emitted VOCs (black), where CCA1 = 22.43% and CCA2 = 15.28%; (**B**) Correlation network depicting main relevant connections (rho ≥ [0.7]) between MALDI-TOF MS ions and identified volatiles.

With the aim to investigate how the alterations produced in profiles of bacterial proteins are reflected in the VOCs distribution, canonical correlation analysis (CCA) and direct correlation analysis using the Pearson method were conducted. The results are showed in Figure 6. The CCA plot (Figure 6A), along its CCA1 and CCA2 axes, was able to explain 37.71% of the variance of a matrix combining MALDI-TOF MS and GC-MS data. This observation points out the possible relation between VOCs emitted by bacteria and their pattern of small proteins. Although it can be understood that this fraction of proteins is not the only factor governing VOCs production, some particular ions seemed to be closely related to the detected volatiles, regardless of the studied strain. In this plot, length and angle of arrows are related to the contribution of a certain variable to the axes. Variables not accompanied by arrows displayed much weaker influence score than others, not being depicted in the automatically scaled graph. The compounds that presented to be the most affected by bacterial ribosomes were dimethyl disulfide, 2-heptanone, 2-undecanol, dimethyl trisulfide, 3-methyl-1-butanol, 1-nonanol, and 2-tetradecanol. While dimethyl disulfide, 2-heptanone, and 1-nonanol can be interpreted as having correlated behavior between them in the three studied bacteria, hexanal and 2-tetradecanol, for example, demonstrated a much more diverse trend than the aforementioned compounds. Figure 6B allows a visual inspection of the nature of the main connections between the two classes of variables. Variables not showed in this network are those that did not present strong or relevant correlation and, in contrast to the last approach, only individual interactions were considered. Interdependency relationships can be also examined: according to the network analysis, the compounds 2-heptanone, 3-methyl-1-butanol, 3-methylbutanoic acid, and dodecanal have their levels associated with many different related proteins—from this, it can be concluded that their expression is associated with intricate factors.

With basis on what was demonstrated concerning the strong correlations between emitted VOCs and protein ions, it was assumed that the levels of such VOCs could provide indirect information about effectiveness of interaction of the bactericidal agent and colonizing bacteria. This approach has potential usefulness in the monitoring of therapeutic response to antimicrobial treatment. Multiple linear regression analysis was conducted in order to create a model based on VOCs as biomarkers of the extension of AgNPs interaction with bacteria. Level of interaction was used as criterion, using 100-SSS*100 as parameter, since it is related to the percentage of modification of unstressed bacterial spectra. VOCs’ responses common to all strains were employed as predictors. As a result, Equation (1) was obtained, with R (coefficient of correlation between observed and predicted values) = 0.89, adjusted R square = 0.79, and standard error of estimate = ±4.10. Such parameters indicate good precision of the generated model:

% of agent effectiveness = 42.9 + 1.6 × 10^−5^ × A_dimethyl disulfide_ − 2.8 × 10^−7^ × A_dimethyl trisulfide_ + 5.3 × 10^−6^ × A_2-heptanone_ − 2.9 × 10^−8^ × A_dodecanal_. (1)

## 4. Discussion

The subjects of our work were: *Escherichia coli* (Gram-negative bacterium) and salivary *Klebsiella oxytoca* (Gram-negative bacterium) and *Staphylococcus saccharolyticus* (Gram-positive bacterium). The differences between cell wall structure of Gram-positive and Gram-negative bacteria are essential in the mechanism of interaction of silver nanoparticles with the surface and the inner part of bacteria. Combined effects of AgNPs and antibiotics (ampicillin, kanamycin, erythromycin, and chloramphenicol) were investigated in the Fayaz et al. (2010) study for bactericidal activity against test strains using the disc diffusion method with Mueller Hinton agar plates [36]. In another work, silver colloid nanoparticles synthesized by reduction of [Ag(NH_3_)_2_]^+^ complex cation by four saccharides revealed high antimicrobial and bactericidal activity against both Gram-positive and Gram-negative bacteria, including *E. coli* and methicillin-resistant *S. aureus*. Authors claimed that the size of particles and very low concentration of silver are crucial to exhibit bactericidal properties of these nanoparticles [37]. In Krishna et al. (2015), antibacterial activity of silver nanoparticles was tested against the two pathogenic strains *Salmonella typhi* and *Salmonella paratyphi* by well-diffusion method. Complex of AgNPs-ofloxacin demonstrated the augmented effect in comparison to separated components [38]. The example of work showing superiority of Gram-positive bacteria over Gram-negative ones in the meaning of resistance against AgNPs is described by Fayaz et al. (2010).

The tested bacteria from this study were two Gram-positive cocci and two Gram-negative rods, including *E. coli*. The antibacterial activity for all antibiotics (especially ampicillin) increased in the presence of AgNPs against test strains. The minimum inhibitory concentration (MIC) evaluated for synergistic effect of AgNPs and antibiotics showed increased less significant influence on growth of Gram-positive bacteria than on Gram-negative species. Cell wall of Gram-positive bacteria is characterized by a thick peptidoglycan layer (~20–80 nm), whereas in Gram-negative bacteria, the peptidoglycan layer is thinner (~7–8 nm) and sandwiched between two layers of periplasmic space and covered by an outer membrane composed by liposaccharides (LPSs) portion. The Gram-negative wall is considered as more susceptible than the Gram-positive one, because the negative charges liposaccharides are attracted toward the positive relative charge available on silver nanoparticles. Moreover, Fayaz et al. (2010) described also negatively charged AgNPs which can attack this type of bacteria by metal depletion [36]. It was found that *E. coli* cells are more prone to increased permeability after ethylenediaminetetraacetate disodium (EDTA) treatment. Liberation of LPS molecules from outer cell membrane are caused by higher metal depletion which weakens maintaining the assembly of the LPSs in membrane [39]. On the contrary, Gram-positive bacteria with thicker layer of peptidoglycan are more resistant to AgNPs because of hindered penetration of nanoparticles and fewer anchoring points for the AgNPs. The layer is highly composed of rigid structure of linear polysaccharide cross-linked by short peptides [36]. The AgNPs-ampicillin complex prevents unwinding of cellular DNA by preferential binding of silver atoms with cells’ DNA [40].

Our work showed that Gram-positive *S. saccharolyticus* was the most resistant strain at all tested AgNPs levels against the remaining Gram-negative ones, when the highest incubation time (12 h) is considered (Figure 5). However, at shorter incubation times *E. coli* and *K. oxytoca* appeared to be more insusceptible for AgNPs influence as reflected in protein profiles acquired by MALDI-TOF MS. After 12 h, bacterial strains exhibited behavior more similar to unstressed ones, deprived of the effect of nanoparticles. Another observation was the fact that both Gram-negative bacteria were more resistant at medium concentration (50 µg mL^−1^) of AgNPs. Such remark was not noticed for *S. saccharolyticus* strain, making this bacterium more distinctive. In recent work of Al-Sharqi et al. (2019), authors investigated antibacterial efficiencies of photoactivated and not-enhanced AgNPs against *E. coli* and *S. aureus*. Cells not treated with silver nanoparticles served as controls. The results for unmodified *E. coli* show that after 24 h of treatment with 12.5 µg mL^−1^ AgNPs, bacterial growth was observed to be 93.33% ± 2.88%. Along with increasing concentration of AgNPs, the microbial number decreased significantly reaching the lowest value of survival viability in the level of 76% ± 1.73% for 100 µg mL^−1^ [41]. Our work confirms that with long time of incubation, *E. coli* is more susceptible for AgNPs influence with increasing level of added stressing agent, from 12.5 to 100 µg mL^−1^.

Protein patterns were processed to obtain heatmap and hierarchical cluster analysis (Figure 4A) that allowed to distinguish three separate clusters belonging to each bacterial strain. It proves effectiveness and appropriateness of the conducted research and shows potential applicability in differentiation purposes. Using lysozyme-stabilized silver nanoparticles, Ashraf et al. (2014) were able to differentiate some of the strains (two *Salmonella enterica*, two *Klebsiella pneumoniae*, four *P. aeruginosa*, two *E. coli*) within the same bacterial species based upon the difference of their antimicrobial activity [42].

The bactericidal effect of silver nanoparticles consists in several mechanisms; (1) AgNPs can adhere onto the surface of cell wall, resulting in membrane damage and altered transport activity [43]. Penetration of cell membrane causes the increased permeability and death of the cell [44]. The damage to membranes is the result of the formation of irregular-shaped pits in the cell surface due to release of LPS molecules [39,45]. (2) Anchoring of silver to cell membrane also inhibits cell wall formation [44,46]. (3) AgNPs modulate cellular signal system and induce oxidative stress caused by generation of reactive oxygen species (ROS) and free radicals [46]. These free radicals can increase porosity of the cell membrane, leading to damage and cell death [44]. (4) AgNPs can be the source of silver ions released by themselves. These Ag^+^ are able to react with thiol groups of enzymes and proteins causing inactivation of them [44,45]. (5) Silver nanoparticles tend to react also with phosphorus-containing compounds such as DNA and prevent its replication as well as cell division and respiratory chain processes [44,45,47]. (6) AgNPs modulate cellular signaling by dephosphorylation of protein substrates in bacteria, resulting in inhibition of cell growth [48]. Finally, changes in nucleoid of bacteria are leading to DNA fragmentation, cell cycle arrest, apoptosis, and inflammatory response [49]. Silver nanoparticles are characterized by many physico-chemical parameters affecting their antimicrobial effectiveness, such as shape, size, concentration, particle dispersion state, and surface charge [43,49].

MALDI-TOF MS technique is often used to identify bacterial strains in timesaving manner and pure culture isolation, such as model organism *E. coli* [50] and other Gram-positive, Gram-negative, anaerobic bacteria and mycobacteria [51,52]. In our work, we applied MALDI-TOF MS technique to obtain protein profiles of bacteria after treatment with silver nanoparticles. In the work of Lok et al. (2006), proteomic analyzes using two-dimensional gel electrophoresis (2-DE) and MS identification revealed that short exposure of *E. coli* cells to AgNPs resulted in alterations in the expressions of a panel of envelope proteins and heat shock proteins (like 30S ribosomal subunit S6) [50]. Twenty protein patterns were identified by MALDI-TOF MS technique from the set of recombinant, human cDNA expression products, together with native proteins isolated from crude mouse brain extracts [53]. Peptide mass fingerprint (PMF) searching of databases appeared to be a cost-effective solution for protein identification purposes. Sixty-two proteins in unsequenced parasitic nematodes were identified by MALDI-TOF MS after evaluation of expressed sequence tag (EST) datasets [54]. This technique was also used, for example, to characterize proteomic patterns in serum from ovarian cancer and healthy control group [55].

MALDI-TOF MS was a suitable technique to investigate binding bare and carbonate-coated AgNPs to the purified tryptophanase (TNase) fragments from *E. coli* to characterize the effect of surface modifications on the binding. It was showed that binding of the protein to AgNPs may sterically block access to the active site caused by lost enzymatic activity of TNase [56]. Using MALDI mass spectra, authors could evaluate the TNase’ enhanced affinity for AgNPs, similarly to our present experiments with protein patterns. Metabolic alterations in bacteria after supplementation with nanoparticles were the subject of interest in the study of Gopal et al. (2013) [57]. They examined the interaction of five types of nanoparticles (Ag, NiO, Pt, TiO_2_, and ZnO) with *P. aeruginosa* and *S. aureus* and postulated two mechanisms of interaction of the above-mentioned NPs with these two pathogenic bacterial strains. The approach consisting in addition of AgNPs at 0 h of bacterial growth, and incubation with bacteria when they grow in the medium, enabled effective interaction of *P. aeruginosa* with unmodified NPs. For *S. aureus*, NPs were added to fully grown bacterial cultures and incubated—a method similar to the one used in our work. In this case, in MALDI-MS spectra, many bacterial peaks were observed. The changes of proteomic profiles were also the object of the researcher interest in the work of He et al. [58]. They carried out the proteomic analysis of graphene-based silver nanoparticles (AgNPs–GE) together with silver nitrate on *P. aeruginosa*. MALDI–TOF/TOF MS was used to identify proteins from bacteria as outcome of 2-DE experiments with silver agents. Authors discovered seven proteins being induced and nine proteins being suppressed by AgNPs–GE.

The effect of the addition of silver nanoparticles on the *S. aureus* strain was again studied in the work of Chudobova et al. (2013) [59]. These researchers conducted a basic characterization of this strain and found out that AgNPs caused considerable inhibition of the growth of *S. aureus*. The authors proposed two methods for determination of silver nanoparticles inhibition effect: using the growth curves and measurements of inhibition zones. Protein fingerprints for the identification of untreated and silver-supplemented *S. aureus* were obtained by MALDI/TOF mass spectra. The usage of the MALDI-TOF MS technique was extended in the more recent work of Chudobova et al. (2015), dedicated to *S. aureus* strain [60]. They focused on examination of the influence of selected heavy metal ions (Ag^+^, Cu^2+^, Cd^2+^, Zn^2+^, and Pb^2+^) at several concentration levels using biochemical methods and mass spectrometry. Additional peaks in silver-fortified bacteria were found in the obtained MALDI-TOF MS spectra demonstrating modifications at the protein level.

The above-mentioned studies are clear examples of how silver ions or nanoparticles may affect the metabolic and proteomic profiles of bacteria, changes in which are visible using the MALDI MS technique. In the present work, a new approach was introduced: MALDI MS and GC-MS data were processed and correlated using statistical methods that allowed to characterize patterns of small proteins and volatile metabolites associated with the interaction of nanoparticles with bacteria.

**Figure 7:**
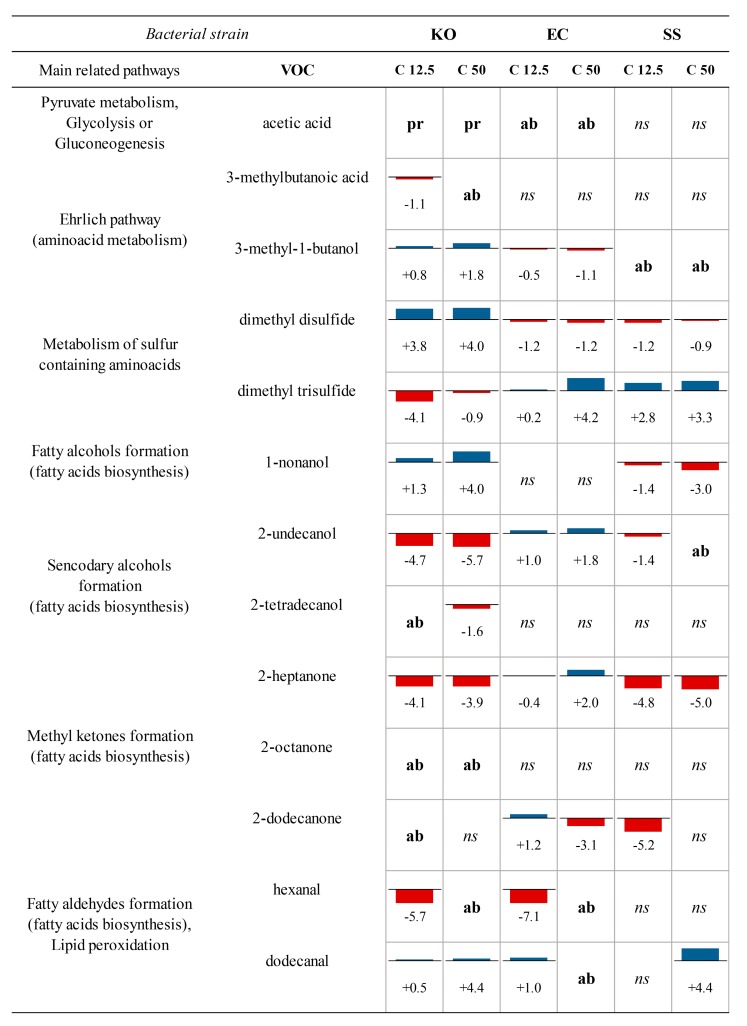
Combination of charts showing altered VOCs and addressed bacterial metabolic pathways, where numbers inside boxes display the base 2 logarithm of fold-change calculated with respect to the response in untreated strain; pr = produced only after AgNPs addition, ab = absent after AgNPs addition, ns = not significant alteration (*p* > 0.05), KO—*K. oxytoca*, EC—*E. coli*, SS—*S. saccharolyticus*, C—concentration (µg mL^−1^), VOC—volatile organic compound [61].

Once it was demonstrated that VOC profiles are able to reflect metabolic alterations in bacterial cells, the effective changes in the response of detected volatiles were evaluated. Figure 7 shows VOCs presenting statistically relevant (*p* < 0.05) difference in their distribution when varying amounts of AgNPs were added to the bacterial culture. It is reasonable to assume that the consequences of the presence of nanoparticles in the medium can vary depending on the examined microorganism. For example, after AgNPs addition, dimethyl disulfide levels increased for *K. oxytoca*, and decreased for the other two bacteria; for dimethyl trisulfide, the other sulfur containing metabolite, an opposite trend is observed.

**Figure 8:**
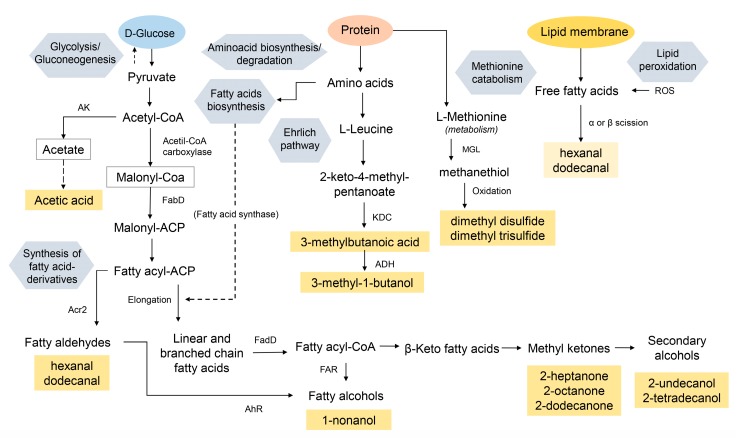
Scheme showing main pathways related to volatile organic metabolites which presented relevant alterations in their responses due to nanoparticles supplementation (AK—acetate kinase; KDC—2-keto acid decarboxylase; ADH—alcohol dehydrogenase; FAR—fatty acid reductase; FabD—malonyl-CoA:ACP transacylase; FadD—fatty acyl-CoA synthase; AhR—aryl hydrocarbon receptor; Acr2—acyl-CoA reductase; MGL—methionine-γ-lyase; ROS—reactive oxygen species).

The origin of volatiles released by three investigated bacteria is mainly a modification of products of the fatty acid biosynthetic pathway, like hydrocarbons, alcohols, and ketones (Figure 8) [61].

Volatile organic compounds secreted from *E. coli* were the object of interest of several researchers who used multiple techniques to investigate the headspace of bacteria. Tait et al. (2014) [62] performed optimization of conditions of HS-SPME-GC-MS analysis of *E. coli* VOCs and evaluated the effect of a culture medium type, SPME fiber type, and the employed GC column on the obtained results. Furthermore, volatile profiles of *E. coli* were investigated in the work of Umber et al. (2013) [63] who found elevated levels of such compounds as: dimethyl disulfide, dimethyl trisulfide, and 1-propanol in the headspace of bacteria in LB broth. These volatiles were not observed in chromatograms obtained from *E. coli*/blood mixture. In our research, we detected these three compounds both in the untreated and AgNPs-supplemented strain of *E. coli*.

VOCs from *K. oxytoca* and other species were studied using ion-molecule reaction–mass spectrometry (IMR-MS) by Dolch et al. (2012) [64]. This method allowed to conduct a short analysis time of 3 min per vial and showed the possibility of process automation. For identification of human pathogenic bacteria (like *E. coli* and *K. oxytoca*), Jünger et al. 2012 used multi-capillary column–ion mobility spectrometry (MCC-IMS). Comparisons of volatilomes were confirmed by thermal desorption–gas chromatography–mass spectrometry (TD-GC-MS). VOC patterns from bacteria enabled fast and cost-effective discrimination of investigated pathogens [65]. Another technique employed for volatile extraction from *E. coli*, *K. oxytoca,* and *P. aeruginosa* was a static head-space-sampler (SHS) coupled to a sensory perception system (SPS). Carrillo and Durán 2019 used this method for distinguish polluted water samples after statistical approaches such as pattern recognition techniques [66].

*S. saccharolyticus* is a bacterial strain responsible for, inter alia, the production of volatile compounds in spoilt mango fruits. GC-MS analyzes revealed the presence of eleven and sixteen volatile organic constituents in healthy and spoilt ripe mango fruits, respectively [67]. There are no other studies concerning volatile profiling of *S. saccharolyticus*. In recent work of Brüggemann et al. (2019), *S. saccharolyticus* isolates from blood cultures and prosthetic joint infection specimens were detected and their genomes were sequenced and analyzed. They found that the closest relative of *S. saccharolyticus* is *Staphylococcus capitis* with an average nucleotide identity of 80%. Moreover, *S. saccharolyticus* manifests host tissue-invasive potential and is associated with prosthetic joint infections [68].

## 5. Conclusions

The reported results confirm that the MALDI-TOF MS technique is an appropriate tool for investigating the influence of silver nanoparticles on metabolism of selected bacterial strains. The comparison between spectra acquired from untreated and silver-enhanced bacteria revealed that addition of AgNPs led to significant changes in their metabolic profiles. Such metabolic alterations were attested by distinguished variations in the emitted bacterial metabolites assessed using GC-MS.

It was demonstrated that interaction between AgNPs and bacteria is deeply associated to the concentration of nanoparticles, incubation time, and strain nature, considerations to be evaluated for the employment of such species as bactericidal agent in medical practice.

Statistical approaches performed in this work reinforced the relation between ribosomal profiles and strain identity, besides that, combination between the variation in protein ion data and VOC patterns allowed the observation of alterations in bacterial inner part closely related with produced volatile metabolites.

Moreover, it can be concluded that the usage of molecular profiles is a promising tool in monitoring of bactericidal performance during infection treatment and to investigate the associated biochemical mechanisms.

## Supplementary Materials

The following are available online at https://www.mdpi.com/2077-0383/8/11/2024/s1, Figure S1: Growth curves of three bacteria cultured in M9 medium. Figure S2: MALDI-TOF MS blanks obtained for (A) M9, MH and TSB media using “DIRECT” protocol and for (B) MH medium after “EXTRACTION” method. Figure S3: Representative (from triplicate experiment) MALDI-TOF MS spectra of E. coli in M9, MH and TSB media obtained using “DIRECT” procedure.
