# Supplementary material for: Monitoring of Bactericidal Effects of Silver Nanoparticles Based on Protein Signatures and VOC Emissions from Escherichia coli and Selected Salivary Bacteria
Source: J Clin Med. 2019 Nov 19;8(11):2024. doi: 10.3390/jcm8112024 (PMC6912796; doi:10.3390/jcm8112024)
Supplement: Supplementary file 1 [file jcm-08-02024-s001.zip › Supplementary Materials/Detailed information regarding data analysis.docx]

R is a programming language that can be executed using a free computing environment, which has many applications in statistical analysis and data visualization [1]. Functions coded in R language are compiled in the so-called “packages”. Installed packages can be accessed by activating the directory “library”. Functions codes are written and freely shared by specialized members of R community [2].

Table S1 specifies input format used in the present work for each type of data analysis / statistical approach and packages employed.

Table S1- Detailed information regarding data analysis

| Method | Software | Input format * | Package specification | Reference | Documentation URL |
| --- | --- | --- | --- | --- | --- |
| GC-MS data processing | R (RStudio console) | GC-MS raw data (.CDF) | xcms, functions ‘xcmsSet’, ‘group’, ‘retcor’, ‘peakTable-methods’ - performs processing of chromatographic data by the execution of a flowchart containing functions which allows peak identification from raw data, peak grouping and retention time correction, generating peak databases | [3] | [4] |
| Principal components analysis (PCA) | IBM SPSS Statistics v.24 | MALDI-TOF-MS ions database - binary format  (.xlsx) | - | - | - |
| Spectrum similarity score (SSS) calculation | R (RStudio console) | MALDI-TOF-MS raw data (.mzXML) | OrgMassSpecR, function ‘SpectrumSimilarity’ -calculates the mass spectral similarity score between two spectra using cos θ = (υ·ν) / [(υ·υ)^1/2^·(ν·ν) ^1/2^] are the intensity vectors, aligned between the two spectra | [5] | [6] |
| Heatmap associated to hierarchical cluster analysis | R (RStudio console) | MALDI-TOF-MS ions database  (.xlsx);  GC-MS peak table (.xlsx) | gplots, function ‘heatmap.2’ – creates a color map according to numeric entries, reorder columns and rows according to selected unsupervised grouping method (Kendall, Sperman or Pearson method), adds respective dendrograms. | [7] | [8] |
| Canonical correlation analysis (CCA) | R (RStudio console) | MALDI-TOF-MS ions database (incidence > 5) (.xlsx) + GC-MS (fold-change of relevant features) (.xlsx) | vegan, function ‘cca’ - executes correspondence analysis, correlating two sets of variables by orthogonal linear combinations. | [9] | [10] |
| Network analysis | R (RStudio console) | Network analysis nodes (.xlsx) + Network analysis edges (.xlsx) | igraph, function ‘plot’ - plots a combination between “nodes” and “edges” inputs, allowing network graphical visualization and edition. | [11] | [12] |
| Multiple linear regression | IBM SPSS Statistics v.24 | GC-MS (fold-change of relevant features) (.xlsx) | - | - | - |

* Input specification:

- GC-MS peak table: retention time *vs.* area for extracted ion chromatogram (EIC) after subtraction of response in medium blanks; for missing values, entry = 0 is set.
- MALDI-TOF-MS ions database: detected ions *vs.* area; for missing values, entry = 0 is set.
- MALDI-TOF-MS ions database - binary format: detected ions *vs.* area, if value > 0, entry =1 is set; for missing value, entry = 0 is kept.
- MALDI-TOF-MS ions database (incidence > 5): database containing only ions which incidence in the data set was superior to 5.
- GC-MS (fold-change of relevant features): compounds which presented relevant changes in their abundances after AgNPs treatment (p value<0.05) were selected and expressed in terms of fold-change (fold-change = average compound area after AgNPs addition / average compound area in unstressed bacteria).
- Network analysis nodes: contains the nodes of the network (protein ions and VOCs). In this set, only variables that presented high correlation (Spearman coefficient > ± 0.7) were considered. This sheet contains 5 columns, id= identification code, name= name of the ion or VOC, input.type = input code (1 for ion, 2 for VOC), type.label = input label (Ion or VOC), incidence.size= number of times that this variable occurred in the database.
- Network analysis edges: contains the edges of the network (connection lines between protein ions and VOCs). In this set, only variables that presented high correlation (Spearman coefficient >0.7) were considered. This sheet contains 5 columns, from = node originating the link, to = link acceptor node, type= type of correlation (positive or negative), weight = set as 2 if the correlation coefficient is between 0.7 and 0.9 (strong correlation), set as 3 if the correlation coefficient exceeds 0.9 (very strong correlation).

References:

[1] R Core Team (2019). R: A language and environment for statistical computing. R Foundation for Statistical Computing, Vienna, Austria. Available in: <https://www.r-project.org/>. Accessed November 11, 2019.

[2] Garrett Grolemund. Quick list of useful R packages (2019). RStudio support. Available in: <https://support.rstudio.com/hc/en-us/articles/201057987-Quick-list-of-useful-R-packages>. Accessed November 11, 2019.

[3] Smith, C.A., Want, E.J., O'Maille, G., Abagyan,R., Siuzdak and G. (2006). “XCMS: Processing mass spectrometry data for metabolite profiling using nonlinear peak alignment, matching and identification.” *Analytical Chemistry*, **78**, pp. 779–787.

[4] Package ‘xcms’:

<https://bioconductor.org/packages/devel/bioc/manuals/xcms/man/xcms.pdf>

[5] Nathan Dodder (2017). OrgMassSpecR: Organic Mass Spectrometry. R package version 0.5-3. Available in: <https://CRAN.R-project.org/package=OrgMassSpecR>. Accessed November 11, 2019.

[6] Package ‘OrgMassSpecR’:

<https://cran.r-project.org/web/packages/OrgMassSpecR/OrgMassSpecR.pdf>

[7] Gregory R. Warnes, Ben Bolker, Lodewijk Bonebakker, Robert Gentleman, Wolfgang Huber Andy Liaw, Thomas Lumley, Martin Maechler, Arni Magnusson, Steffen Moeller, Marc Schwartz and Bill Venables (2019). gplots: Various R Programming Tools for Plotting Data. R package version 3.0.1.1. Available in: <https://CRAN.R-project.org/package=gplots>. Accessed November 11, 2019.

[8] Package ‘gplots’:

<https://cran.r-project.org/web/packages/gplots/gplots.pdf>

[9] Jari Oksanen, F. Guillaume Blanchet, Michael Friendly, Roeland Kindt, Pierre Legendre, Dan McGlinn, Peter R. Minchin, R. B. O'Hara, Gavin L. Simpson, Peter Solymos, M. Henry H. Stevens, Eduard Szoecs and Helene Wagner (2019). vegan: Community Ecology Package. R package version 2.5-5. Available in: <https://CRAN.R-project.org/package=vegan>. Accessed November 11, 2019.

[10] Package ‘vegan’:

<https://cran.r-project.org/web/packages/vegan/vegan.pdf>

[11] Csardi G, Nepusz T: The igraph software package for complex network research, InterJournal, Complex Systems 1695. 2006. Available in: <http://igraph.org>. Accessed November 11, 2019.

[12] Package ‘igraph’:

<https://cran.r-project.org/web/packages/igraph/igraph.pdf>
