# Supplementary figures and images for: Monitoring of Bactericidal Effects of Silver Nanoparticles Based on Protein Signatures and VOC Emissions from Escherichia coli and Selected Salivary Bacteria
Source: J Clin Med. 2019 Nov 19;8(11):2024. doi: 10.3390/jcm8112024 (PMC6912796; doi:10.3390/jcm8112024)

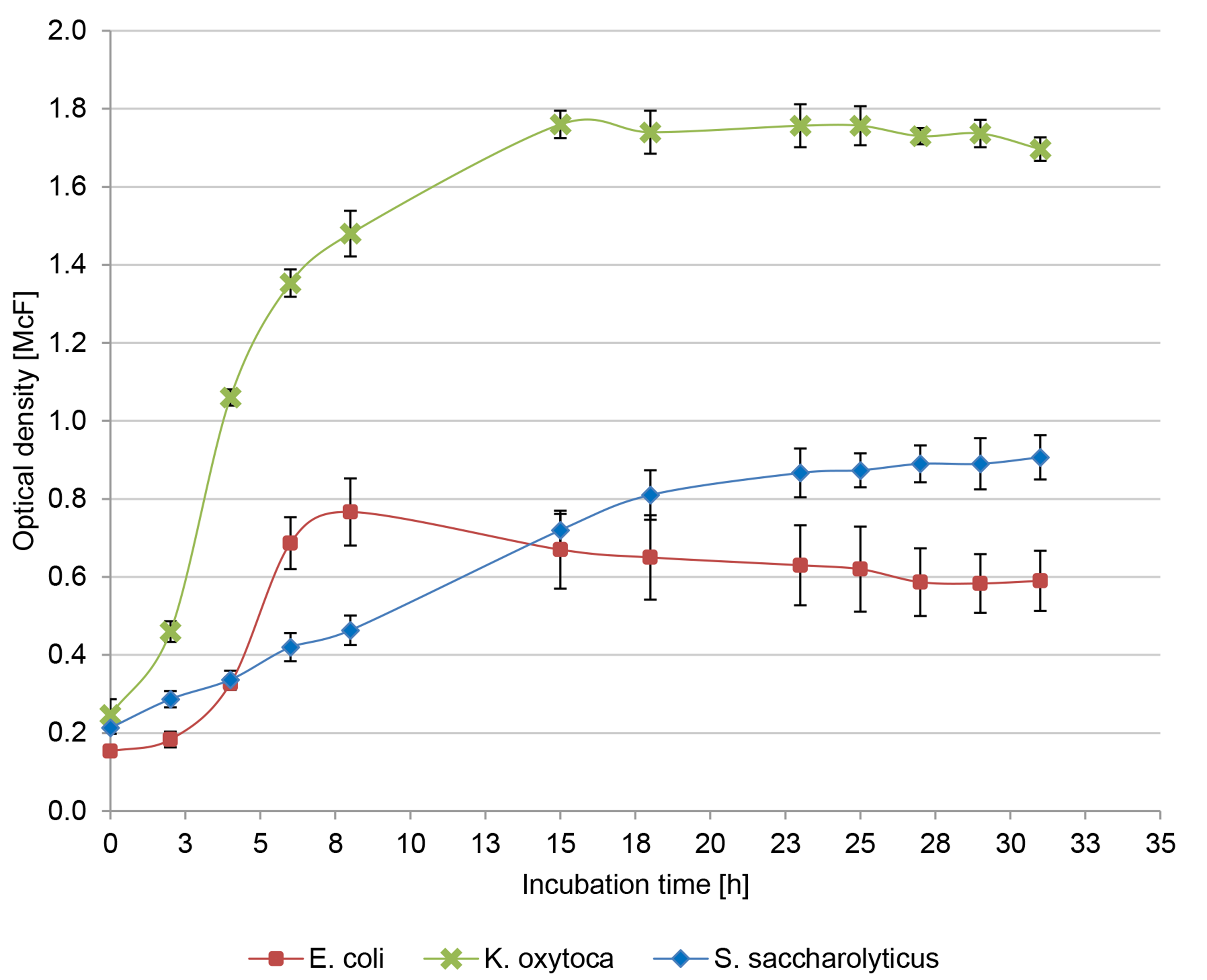

Supplement: Supplementary file 1 [file jcm-08-02024-s001.zip › Figure S1.tif]

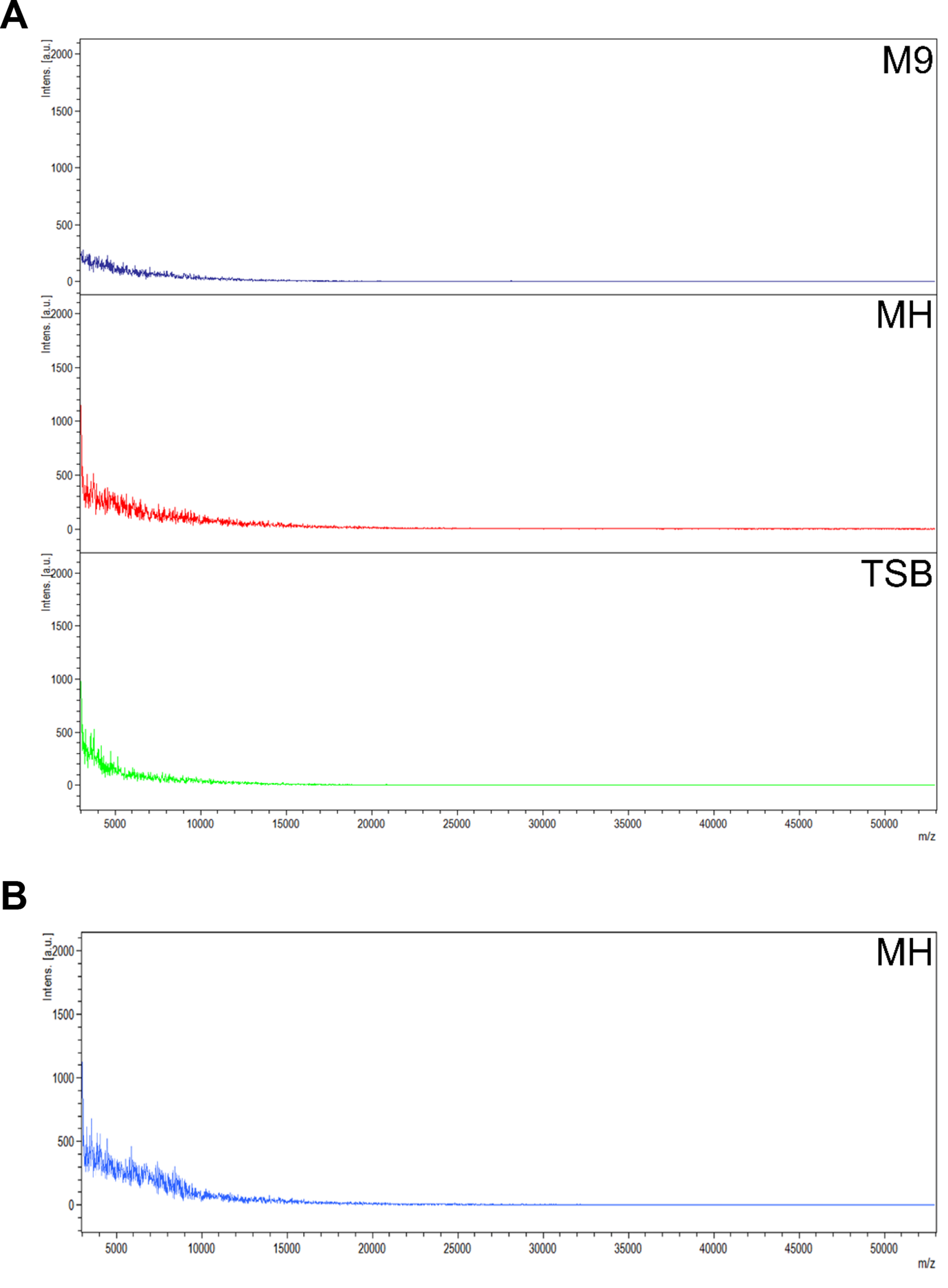

Supplement: Supplementary file 1 [file jcm-08-02024-s001.zip › Figure S2.tif]

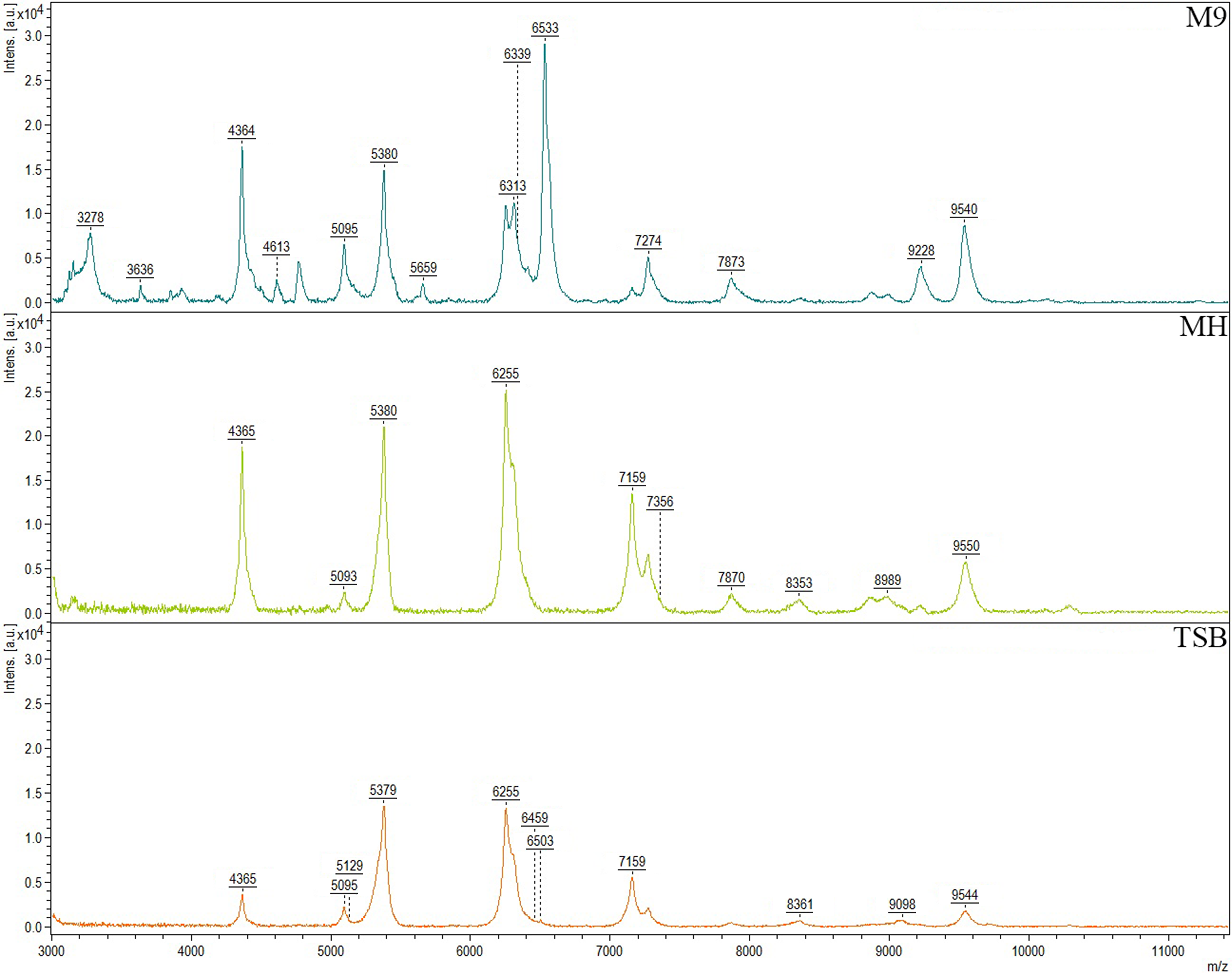

Supplement: Supplementary file 1 [file jcm-08-02024-s001.zip › Figure S3.tif]
